# MicroRNA-Mediated Control of Inflammation and Tolerance in Pregnancy

**DOI:** 10.3389/fimmu.2019.00718

**Published:** 2019-04-05

**Authors:** Ranjith Kamity, Surendra Sharma, Nazeeh Hanna

**Affiliations:** ^1^Women and Children Research Laboratory, Division of Neonatology, Department of Pediatrics, NYU Winthrop Hospital, Mineola, NY, United States; ^2^Department of Pediatrics, Women and Infants Hospital, Warren Alpert Medical School of Brown University, Providence, RI, United States

**Keywords:** microRNA, immune tolerance, endotoxin tolerance, extracellular vesicles, pregnancy, maternal-fetal interface, innate immunity

## Abstract

Gestational age-dependent immune intolerance at the maternal-fetal interface might be a contributing factor to placental pathology and adverse pregnancy outcomes. Although the intrauterine setting is highly choreographed and considered to be a protective environment for the fetus, unscheduled inflammation might overwhelm the intrauterine milieu to cause a cascade of events leading to adverse pregnancy outcomes. The old paradigm of a sterile intrauterine microenvironment has been challenged, and altered microflora has been detected in gestational tissues and amniotic fluid in the absence of induction of significant inflammation. Is there a role for endotoxin tolerance at the maternal-fetal interface? Endotoxin tolerance is a phenomenon in which tissues or cells exposed to the bacterial product, particularly lipopolysaccharide, become less responsive to subsequent exposures accompanied by decreased expression of pro-inflammatory mediators. This could also be related to trained or experienced immunity that leads to the successful outcome of subsequent pregnancies. Adaptation to endotoxin tolerance or trained immunity might be critical in preventing rejection of the fetus by the maternal immune system and protecting the fetus from excessive maternal inflammatory responses to infectious agents; however, to date, the exact mechanisms contributing to the establishment and maintenance of tolerance at the maternal-fetal interface remain incompletely understood. There is now extensive evidence suggesting that microRNAs (miRNAs) play important roles in the maintenance of a healthy pregnancy. miRNAs not only circulate freely in extracellular fluids but are also packaged within extracellular vesicles (EVs) produced by various cells and tissues. The placenta is a known, abundant, and transient source of EVs; therefore, our proposed model suggests that repeated exposure to infectious agents induces a tolerant phenotype at the maternal-fetal interface mediated by specific miRNAs mostly contained within placental EVs. We hypothesize that impaired endotoxin tolerance or failed trained immunity at the maternal-fetal interface will result in a pathological inflammatory response contributing to early or late pregnancy maladies.

## Introduction

Our current understanding of immunity at the maternal-fetal interface has been shaped by acceptance of periodic concepts and approaches formed based on tissue-allograft studies in non-pregnant individuals, male or female, and by the paradigm suggesting the uterus as a sterile organ (1, 2). However, our contemporary understanding of the role of the placenta, hormones, and novel cellular controls warrants re-evaluation of old paradigms associated with fetal immune-protection in both hostile and normal intrauterine microenvironments (3, 4). The decidua, the maternal tissue in pregnancy, is replete with immune cells that should intrinsically harm the fetus; however, normal pregnancy ensues, suggesting that the placenta transforms these cells into a specialized, pregnancy compatible milieu (5). What remains incompletely understood is why the placenta fails to counter challenges from intrauterine infections or sterile inflammation. This is further complicated by the observations that some women can experience a successful pregnancy despite repeated exposure to infectious agents, particularly endotoxins, and other environmental factors. This review discusses recent observations that might explain successful pregnancy outcomes despite repeated intrauterine infections and potentially provide important insights into the role of the placenta via secretion of extracellular vesicles (EVs).

Despite constant exposure to hygienic challenges and infection during pregnancy, most of the 4 million annual deliveries in the United States have a successful outcome without presenting any clinical evidence of intrauterine infection (6). In this regard, the onus can be placed on the placenta to counter inflammation and ensure trained immunity for a successful pregnancy (7–10); however, it remains unclear how the placenta programs these pregnancy compatible events. The placenta is a transient organ that supports fetal growth and development by ensuring respiratory gas exchange, regulates maternal-fetal nutrient transport, provides protection for the fetus against maternal immunity, and acts as a transient endocrine organ by producing hormones, such as estrogen, progesterone, human chorionic gonadotropin, and human placental lactogen, while protecting the fetus from external infectious and immune threats (11, 12). Increasing evidence suggests that molecular exchanges occur between maternal and fetal systems to enable adaptation of maternal physiology for growing fetal requirements during gestation, with the focus placed on placenta-derived EVs as a medium of placental communication with maternal physiology (13–15). An important question arises from these observations and concerns whether placental EVs play a role in protecting the fetus from infectious agents.

Among various models of immune tolerance, microRNAs (miRNAs) are implicated as mediators, as well as markers of immune tolerance in various tissues; however, the mechanisms associated with miRNA-mediated immune tolerance in gestational tissues are not well-understood. Because miRNAs are also included as cargo in EVs, do placental EVs and their embedded miRNA cargo play a role in immune tolerance at the maternal-fetal interface? This review focuses on immune tolerance at the maternal-fetal interface and reviews the role of miRNA in mediating immune tolerance via EVs in gestational tissues.

## Maternal-Fetal Interface Immunity and Its Role in Adverse Pregnancy Outcomes

Pregnancy is a period of physiological stress accompanied by a vital balance between proinflammatory and anti-inflammatory stimuli. Disruption of this delicate balance at the maternal-fetal interface has been linked to various adverse pregnancy outcomes. Starting from exposure to seminal antigens at coitus, to implantation, an active inflammatory period facilitates implantation and initial pregnancy (16). The cytokines and chemokines in seminal fluid play a role in attracting regulatory T cells (Tregs) to the microenvironment (17). Female dendritic cells recognize fetal antigens and cross-present seminal-fluid antigens that transform effector CD4^+^ T cells into Tregs, which are then recruited to the endometrium. Tregs and decidual natural killer (dNK) cells increase during early pregnancy and play a vital role in implantation and maintenance of pregnancy (17–19). Although *in vitro* fertilization (IVF) pregnancies involving fertilized eggs with washed semen do occur, Treg recruitment to the implantation site appears to be a physiologically helpful and critical event for implantation and pregnancy maintenance (20, 21). dNK cells and Tregs are integral cellular components that contribute to normal placentation and vascular support at the maternal-fetal interface, while playing an important role in preventing fetal rejection (22).

This period of inflammatory activity during implantation is subsequently followed by a period of relative inflammatory quiescence during mid-pregnancy to enable fetal growth and development (17, 23). Dysregulated immunity with excessive maternal inflammation further into pregnancy contributes to impaired angiogenesis, especially of the spiral arteries, resulting in gestational vascular disorders, such as preeclampsia (24–27). The concept of trained immunity and inflammation tolerance might be applicable in the case of preeclampsia, as subsequent pregnancy with the same partner within a few years of first pregnancy is not always associated with recurrence of the pregnancy complication (28, 29).

Pregnancy can be divided into three phases of inflammatory milieu: implantation (inflammatory), active gestation (anti-inflammatory), and parturition (inflammatory) (17, 23). At parturition, labor is induced by an inflammation-associated cascade of events that result in delivery. Premature activation of this process results in preterm labor and delivery. Why are the placenta or its EVs and miRNAs unable to blunt this premature activation of inflammation? It is possible that inflammation tolerance (not necessarily immune tolerance) escapes the placenta due to detrimental activation of dNK cells and Tregs, or that EVs carry an inflammatory cargo that does not counter inflammation.

## Types of Inflammation at the Maternal-Fetal Interface: Infection-Mediated or Sterile?

A fetus in the intrauterine environment is believed to be in a relatively protected state. Occasionally, microbes gain passage into the intrauterine milieu by ascending infection or transplacental routes, with the transplacental transmission of infection from mother to fetus capable of occurring antenatally or perinatally (12). Pregnant women with altered immunity secondary to various stressors, including infection, can potentially experience preterm delivery. Romero et al. showed that despite evidence of preterm birth being linked to infection, preterm birth without infection is more common (30). Furthermore, antibiotics alone are ineffective at preventing preterm birth related to infection, suggesting that preterm birth induced by infection is mediated by inflammation rather than the organism *per se* (31). One possible explanation for antibiotic failure is that inflammatory damage has already been established, and detrimental pathways have been initiated and cannot be controlled by poorly timed treatment with antibiotics (32). Our understanding of inflammatory signals also suffers from a poor definition of sterile inflammation and its role in programming preterm birth. Therefore, it is important to understand the mechanistic differences between sterile inflammation and infection-mediated inflammation.

Pro-inflammatory stimuli can arise from both host (self) and alien (non-self) sources, also described as “danger” and “stranger” stimuli (23), that include damage-associated molecular patterns (DAMPs; also known as alarmins) and pathogen-associated molecular patterns (PAMPs), which interact with a group of pattern-recognition receptors (PRRs) expressed on the cell surface. Sterile pathways that trigger inflammation include host factors (e.g., tissue injury, cell death, and environmental factors, such as low oxygen tension and elevated uric acid) that act via DAMPs (25). These differ from infectious triggers (e.g., bacteria or viruses) that act via PAMPs (23). However, pathological inflammatory processes can be triggered by either sterile or infection-mediated pathways.

DAMPs are a group of endogenous intracellular molecules released in the early stages of unplanned cell death to signal cell injury (33). The most common DAMPs include uric acid, high-mobility group box 1, interleukin (IL)-1α, and cell-free DNA. High-mobility group box 1 levels increase in multiple animal models along with sterile inflammation, as well as in human models of acute organ injury, autoimmune diseases, and cancer (34–37). High concentrations of uric acid, a byproduct of cell death, can form monosodium urate crystals in the presence of extracellular sodium and induce acute inflammation. Upon exposure to foreign antigens and as the non-specific first line of defense against foreign microorganisms, monocytes differentiate into macrophages capable of phagocytosing the offending agents. Monosodium urate crystals, upon phagocytosis by antigen-presenting cells, can interact with the NALP3 inflammasome to convert pro-IL-1β to IL-1β, thereby resulting in an inflammatory response (23) recognized as pyroptosis. Inflammasome activation has been shown in both intra-amniotic infections as well as sterile intra-amniotic inflammation with preterm labor (38). Furthermore, treating sterile inflammation by inhibiting inflammasome activation has also been shown to reduce preterm delivery in a mice model (39).

PRRs include Toll-like receptors (TLRs) 1 through TLR13, C-type lectins, scavenger receptors, and nucleotide-binding oligomerization-domain-like receptors, all of which operate transduction pathways resulting in cytokine-mediated inflammatory responses. PRRs are expressed in multiple human cells, including decidua, placenta, membranes, and myometrium, throughout pregnancy and in immune and non-immune cells (23, 40–42). The release of DAMPs secondary to tissue injury from hypoxia-ischemia, oxidative stress, vascular dysfunction, or other stressors is implicated in sterile inflammation that acts not only on the placenta but also on the uterus, cervix, fetal membranes, and the fetus. This inflammatory process needs to be tightly regulated since such inflammation left unchecked can cause extensive tissue injury, septic shock, and death. In the placenta, this resultant maternal-fetal inflammation is suggested to contribute to various adverse pregnancy outcomes, including placental dysfunction, preeclampsia, intrauterine growth restriction, and preterm labor (23). Therefore, attenuation of this inflammation at the maternal-fetal interface can play a key role in fetal health and survival.

## Induction of Innate Immunity via microRNAs and Shaping of Immune Tolerance: From Mice to Humans

The systemic immune system is regulated and dominated by T lymphocytes and adaptive immunity. On the other hand, the decidual leukocyte population in the pregnant uterus is replete with NK cells (65–70%) and antigen presenting cells (macrophages and dendritic cells 10–20%), both contributors to innate immunity (4, 5). Other cell types have also been described to a lesser extent at the maternal-fetal interface, in varying numbers at different stages of pregnancy, and in certain pathological conditions (43, 44), including innate lymphoid cells, other T cell subsets and B cells. Innate immunity at the maternal-fetal interface is of a specialized variety, wherein NK cells and macrophages are pregnancy compatible and support local vascular activity. Prolonged exposure to microbial products, such as lipopolysaccharide (LPS) induces a form of innate immunity that resembles trained immunity (memory) and blunts subsequent responses to unrelated pathogens [referred to as endotoxin tolerance] (45). What is the molecular basis of endotoxin tolerance, and does this occur in the female reproductive tract? Recent pioneering work by Seeley et al. (45) suggests that repeated exposure to LPS induces tolerance-associated miRNAs (miR-221 and miR-222) in macrophages, with this tolerance phenotype achieved through silencing of inflammatory genes and chromatin remodeling. Most studies of endotoxin tolerance have been undertaken in mouse models, as well as human cell and tissue models, resulting in links between endotoxin tolerance and protection against tissue injury and death (46, 47). Seeley et al. (45) suggest that in humans with sepsis, increased expression of these miRNAs is associated with immunoparalysis and organ damage; therefore, it is possible that a threshold level of tolerance-associated miRNAs needs to be maintained for a longer period of time in order to influence a better outcome in humans.

The molecular mechanisms associated with endotoxin tolerance are believed to be multifactorial, with multiple levels of negative feedback to blunt the inflammatory response. Mice deficient in IL-10 are more susceptible to endotoxic shock following repeated exposure to LPS, suggesting a role for IL-10 in endotoxin tolerance (48). In gestational tissues, endotoxin tolerance has been identified in mouse models, whereas human studies are lacking. During pregnancy, this adaptation due to tolerance might be critical to preventing fetal rejection by the maternal immune system, as well as protecting the fetus from excessive maternal inflammatory responses to various infectious and inflammatory agents. An increase in proinflammatory cytokine responses to bacterial insult can trigger undesirable consequences, including preterm labor and delivery, as well as fetal mal-development and inflammatory injury. Although bacterial exposure during pregnancy is associated with preterm delivery and intra-amniotic infections (49, 50), a wide array of organisms have been described in the maternal-fetal compartments in healthy pregnancies. Interestingly, the presence of an organism in the uterine environment is not always pathological, as previously reported in gene-amplification studies using amniotic fluid from women who delivered healthy newborns following an uncomplicated pregnancy (32, 51, 52).

## Immune Tolerance Mediated by microRNA

miRNAs are small (18–22 nucleotides), non-coding RNA sequences that play an important role in regulating multiple cellular processes critical for development, differentiation, and organ function and are associated with marked biological consequences in health and disease (53). miRNAs act as negative regulators at the post-transcriptional level by binding to the 3′ untranslated region on target mRNA to inhibit the translation of respective proteins. Thousands of genes are regulated by miRNAs, with the list continuing to grow (53, 54). It is estimated that ~60% of all protein-coding genes can be regulated by miRNA (54); therefore, it is now accepted that mutations that cause dysfunctional miRNA can potentially affect multiple disease conditions.

Multiple organs harbor specific miRNAs that play vital roles. miR-122 is associated with cholesterol and lipid metabolism in the liver (55), whereas the miR-1 and mir-133 families regulate heart development and play roles in cardiovascular disorders (56, 57). Additionally, numerous recently identified miRNAs have been implicated in a wide array of human diseases, including cardiovascular diseases, neoplasms, and liver and kidney disorders (58–60). Moreover, early gestational tissues, such as human blastocysts, express miRNA, which might be essential for successful implantation and subsequent survival by guiding processes that navigate the intrauterine environment. Euploid and aneuploid embryos exhibit differential expression of miRNAs, ultimately resulting in different eventual outcomes (61). Furthermore, the presence of miRNAs in breast milk and serum could represent markers or mediators of cell signaling. Maternal serum miR-191 is currently being investigated as a potential non-invasive candidate for aneuploidy, whereas other miRNAs, including miR-191, miR-372, and miR-645, have been implicated in IVF failure, and elevated levels of miR-25, miR-302c, miR-196a2, and miR-181a have been identified in degenerate embryos as compared with their levels in blastocyst embryos (62).

In addition to regulating multiple cellular processes, various miRNAs are also implicated in regulating the immune system, including the differentiation and function of innate immune cells (63, 64). miRNAs have also been shown to regulate immune responses to bacterial, viral and parasitic infections (65–67). For example, miR146a polymorphism has been linked to increased risk of malaria in pregnant women. Also, miR 221 negatively regulated innate antiviral response, while miR 34/449 family has been implicated in various viral infections [(66), review]. The role of miRNA in bacterial infections is being studied in various scenarios- from a suggested role in Helicobacter pylori and Epstein-Barr virus-induced cancers [(68), review], to pathogen triggered TLR pathway stimulation.

Multiple miRNAs are up-regulated via TLR-ligand stimulation in monocytes (69) and suggested to play a major role in mediating immune tolerance by regulating the TLR pathway through TLR-receptor transcription and/or creating feedback loops by suppressing key downstream molecules that in turn down-regulate TLR activation (70). Moreover, the pattern of miRNA expression is related to the type of TLR ligand involved in its stimulation, overall ligand concentration, and the type of cells being stimulated.

Increases in miR-146a levels in response to LPS stimulation was observed in THP1 cells (63, 69, 71, 72). Additionally, Nahid et al. (71) showed that exposure to high-dose LPS (1,000 ng/mL) increases levels of the inflammatory cytokines tumor necrosis factor (TNF)α, IL-1-receptor-associated kinase (IRAK)1, and TNF-receptor-associated factor (TRAF)6 along with a simultaneous increase in miR146a levels. On the other hand, when the same cells were primed with low-dose LPS (10 ng/mL), transient elevations in TNFα, IRAK1, and TRAF6 were observed along with upregulated miR146a levels, with subsequent exposure to high-dose LPS (1,000 ng/mL) resulting in a blunted TNFα response in the presence of upregulated miR146. Other studies suggested that elevated miR-146a levels are involved in regulating TLR signaling and cytokine production by downregulating the inflammatory response (73, 74). Furthermore, miR-146a induction is mediated by NF-κB via TLR-ligand stimulation (69). Conversely, miRNAs can directly target TLRs in order to create a feedback loop that regulates the inflammatory response, with previous studies reporting that miR-105 targets the mRNA encoding the TLR2 receptor in order to attenuate its translation (75, 76). Additionally, miR-146a targets TRAF6 and IRAK activities to inhibit TLR4 signaling and downregulate NF-κB activation (69). By contrast, miRNAs, such as miR-155, can be significantly downregulated after the induction of endotoxin tolerance, suggesting cross-talk between miRNAs in order to maintain immune homeostasis and suppress proinflammatory responses. In addition to miRNAs, long non-coding RNAs are also altered upon endotoxin challenge and negatively regulate TLR signaling, thereby contributing to immune tolerance.

miRNAs are also being studied as diagnostic and therapeutic tools (77). A recent study suggests that decidual tissue from patients with recurrent spontaneous abortions shows decreased expression of miR-146a-5p (78). Similarly, quantification of 30 miRNAs from peripheral blood taken during the first trimester was used to predict preeclampsia, a late pregnancy complication (79). These observations support further study of miRNAs from gestational tissues and peripheral blood to investigate their role(s) in both endotoxin tolerance and pregnancy complications.

## Placenta and microRNA

The human placenta produces numerous miRNAs. miRNAs play roles in various key steps in pregnancy, including implantation, maintenance, and labor (80–84). miRNAs have been identified in trophoblasts and mostly originated from the two largest clusters on chromosomes 14 and 19 (C14MC and C19MC, respectively). Most miRNAs identified in primary trophoblasts originate from C19MC, which gives rise to 46 intronic miRNAs that are converted to 54 mature miRNAs (85). miRNAs from C19MC are found in human embryonic stem cells and play an important role in cell proliferation, invasion, and differentiation. C19MC expression is reduced in extravillous trophoblasts and several malignancies while the increase in C19MC expression confers resistance to viral infections (85, 86).

Interestingly, abundant expression of miRNA does not always translate to functional significance. A previous study reported the deletion of the miR379/410 cluster (from C14MC) without consequence (84). Additionally, miR-675 exhibits anti-proliferative effects by silencing *insulin-like growth factor receptor-1*, whereas miR-675 deletion is associated with placental overgrowth (87). Moreover, miR-378a-5p and miR-376c are involved in trophoblast proliferation and invasion regulated by the nodal signaling pathway (88, 89), and the miR17-92 cluster regulates primary human trophoblast differentiation (90). Furthermore, recently identified miR-155 inhibits trophoblasts invasion and is implicated in the pathogenesis of preeclampsia, and increased levels of plasma and placental miR-210 have been reported in association with preeclampsia (91). Although additional miRNAs continue to be discovered their precise clinical implications and roles remain elusive; however, a non-invasive sampling of such placenta-derived miRNAs is potentially useful for diagnosing placental dysfunction.

The majority of miRNAs function in the cell of origin by regulating mRNA levels and translation; however, some miRNAs are selectively secreted by cells into the extracellular space (mainly packaged within EVs) to regulate intercellular signaling of distant “target” cells. Analyses of miRNA from EVs show that EVs' miRNAs content is distinct from that found in the cytoplasm of donor cells from which they were derived. This suggests active miRNA sorting into these vesicles (92–95), although the exact mechanism remains unclear. Our unpublished data confirm that placental miRNAs play an important role in placental endotoxin tolerance resulting in blunted immune response to repeated exposure to LPS. Specifically, placenta-derived miR519c (derived from C19MC) was shown to inhibit TNFα gene expression in our placental explant model. miR519c is placenta-specific and produced by trophoblasts and can be released as free miRNA or packaged into EVs.

## Extracellular Vesicles in Gestational Tissues

EVs are membrane vesicles of various sizes that are secreted by almost every cell type and multiple organisms ranging from bacteria to humans. EVs are broadly classified according to size and origin into two different categories: exosomes and microvesicles (MVs; including microparticles and apoptotic bodies) (14, 96). MVs are larger membrane-derived vesicles >150 nm in size and secreted directly from the cell membrane. Exosomes are cell-secreted, membrane-derived nanovesicles, which represent a subpopulation of EVs measuring from 40 to 120 nm and with a density of between 1.13 and 1.19 g/mL (14). Exosomes arise from the endosomal compartment as intraluminal vesicles and are secreted by the fusion of endosomes or multivesicular bodies (MVBs) with the cell membrane. Additionally, exosomes can be described according to morphological characteristics (spherical or cup-shaped) and surface markers (CD63, CD9, CD81, and Tsg101) (14, 97). Various gestational tissues, including pre-implantation embryos, oviduct epithelium, placental trophoblasts, and endometrium, secrete EVs, with their source determined according to cell-specific markers. Previous studies suggest that miRNAs are protected from RNase degradation in serum by encapsulation within EVs, which act as carriers of regulatory RNA (53, 98).

## The Role of Extracellular Vesicles in Normal Pregnancy and Gestational Vascular Disorders

The placenta releases EVs as early as the sixth gestational week, with this activity implicated in regulating maternal pregnancy physiology and fetal development, including pregnancy-induced hypertension, gestational diabetes, preterm labor, and delivery (14, 15, 97, 99). EVs (especially exosomes) play a vital role in the preparatory cross-talk between endometrium and embryo at the onset of pregnancy (83, 100–102). Trophoblast-derived EVs harbor molecules specific to placental physiology and cell-cell communication and that exert diverse effects on maternal and embryonic compartments. These molecules include fibronectin, syncytin, galectin-3, human leukocyte antigen-G, and cytokines as well as bioactive lipids and/or miRNAs, capable of immunomodulation (103). Placental alkaline phosphatase (PLAP), a membrane protein of the placenta, is primarily produced by syncytiotrophoblasts and used as a marker to identify placenta-derived exosomes in maternal circulation (104). PLAP^+^ exosomes have only been described in the peripheral circulation of pregnant women (13, 14), with the number of placental exosomes positively correlated with total exosomes concentrations during the first trimester in normal pregnancy (105). The total number of exosomes (CD63^+^) and placental exosomes (PLAP^+^) present in maternal plasma increases exponentially in the second and third trimesters (14). Although PLAP shows potential as a useful marker for measuring exosomes in normal pregnancy, its levels have not yet been quantified in pathological pregnancies. Furthermore, syncytiotrophoblast EVs are secreted into maternal circulation as early as the tenth gestational week, their numbers increasing by the third trimester; however, their excessive secretion has also been reported in association with preeclampsia (15).

EVs in pregnancy play a pro-coagulant role to a greater degree than that observed in non-pregnant women (103). Levels of EVs harboring the tissue factor antigen, which is involved in the first step of the coagulation cascade, increase during pregnancy, and a significant increase in EVs harboring tissue factor is related to gestational vascular disorders, suggesting a pro-coagulant immune profile in such situations. Moreover, EVs play a significant role in regulating the inflammatory milieu during pregnancy, as EVs from pregnant women show higher levels of inflammatory proteins. Furthermore, EVs from hypoxic trophoblasts exhibit a more intense inflammatory response to peripheral blood mononuclear cells than do EVs from normal trophoblasts. Additionally, placenta-derived EVs carry a functional Fas ligand and TNF-related apoptosis-inducing ligand molecules that convey signals for apoptosis, suggesting a role in establishing EV-mediated immune privilege on behalf of the fetus (106). It is also possible that placental EVs modulate the response to LPS in pregnant women. Unpublished data from our lab showed that exosomal miRNAs mediate endotoxin tolerance in the placenta after repeated LPS exposure. Cytochalasin-D, an inhibitor of exosomes release and uptake, blocked endotoxin tolerance and restored the proinflammatory response in placental explants treated with repeated doses of LPS, thereby suggesting that exosomes mediate endotoxin tolerance in the placenta.

## Extracellular Vesicle Targeting of Specific Tissue and Cells

The process of EV formation is complex and aimed at secreting selectively prepared vesicles with their content and presenting surface markers geared toward specific target cells. Although the mechanisms of exosomes biogenesis and release continue to be investigated, little is known regarding how exosomes' content is regulated as well as which cells they target. Cell-specific markers presented by exosomes are proposed to play a role in target-cell identification and interaction. Adaptor proteins reportedly recruit an exosome-associated helicase (MTR4) to unique RNA substrates, and exosome cofactors, such as the TRAMP-like protein complex, localize to the cytoplasm and recruit exosomes to specific viral RNA for degradation (107). Other examples of EV targets include cancer cells, which subsequently use EVs to target other organs, such as the lung and liver, to identify pre-metastatic niches based on specific integrin composition (108).

## A Placental Model for microRNA-Mediated Endotoxin Tolerance via EVs

**Figure 1:**
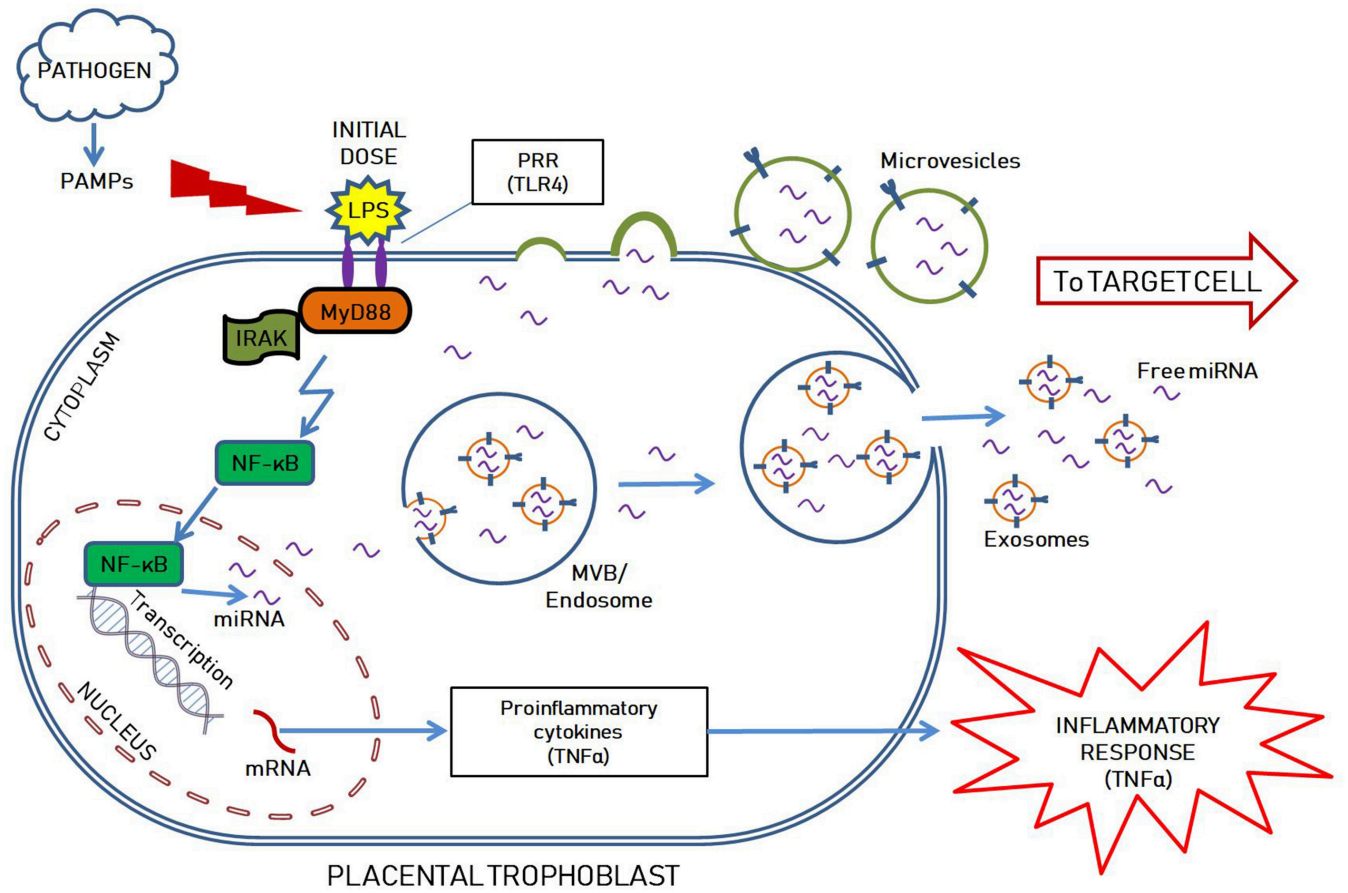
Hypothetical illustration showing LPS mediated inflammatory response, possible mechanism of anti-inflammatory miRNAs mediated endotoxin tolerance as well as packaging within extracellular vesicles (EVs; including exosomes and microvesicles) in a placental trophoblast. The initial LPS dose stimulates TLR4 pathway in the placental trophoblast to prompt the initial inflammatory response, while also upregulating miRNA transcription. The miRNAs are either found freely in the cytoplasm and released outside the cells, or selectively packaged into extracellular vesicles by various mechanisms. Exosomes are formed intraluminally in the endosomal system within multivesicular bodies (MVB) while microvesicles are secreted by membrane derived vesicle formation. They are selectively packaged with nucleic acids (DNAs, mRNAs, miRNAs), proteins, lipids and carbohydrates, specific to the cell of origin and the intended target. The EVs and free miRNAs are transported to the target cell via the extracellular space.

**Figure 2:**
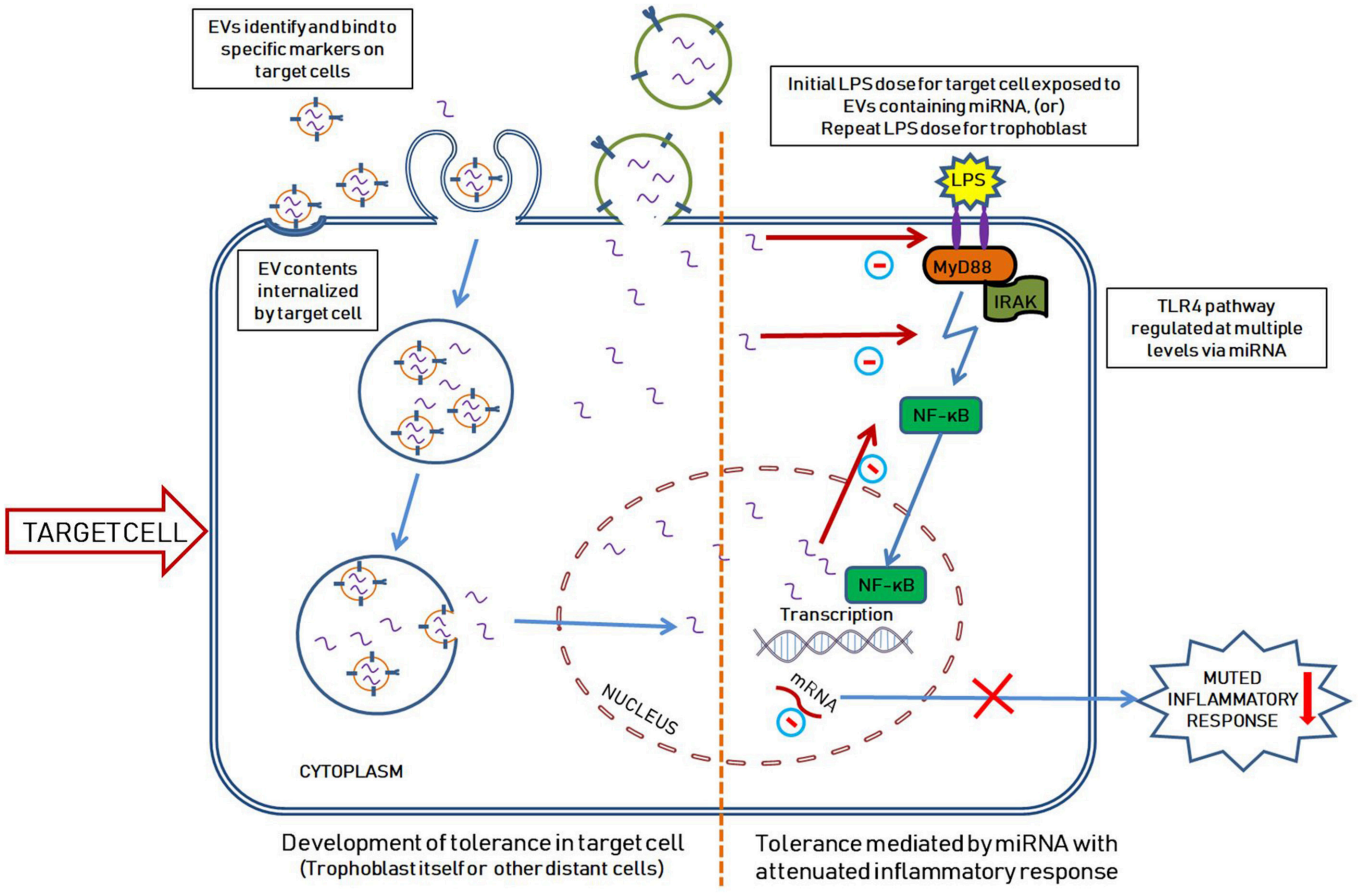
Hypothetical illustration showing the development of endotoxin tolerance in a target cell mediated by miRNA packaged within the EVs. EVs carrying miRNAs (and other contents) are released from the original placental trophoblasts upon exposure to initial LPS dose as seen in Figure 1. The released EVs travel to the target cells where they are selectively internalized by different mechanisms as shown. Microvesicles are internalized into the cytoplasm by fusing with the cell membrane while exosomes are internalized into vesicular bodies, lipid rafts on cell surface or by directly binding with specific cell surface receptors (96). The target cell in this scenario can represent a distant cell (exposed to first dose of LPS) or the original trophoblast cell (exposed to a second dose of LPS). We hypothesize that the miRNAs within the EVs are released into the cytoplasm and enter the nucleus thereby negatively regulating the inflammatory pathway at multiple levels (including direct inhibition of TLR4, downregulating mRNA translation of TLR and negative feedback for NF-κB mediated TLR4 pathway). Upon exposure to a second dose of LPS, the original tolerized cell generates an attenuated inflammatory response (since it has high levels of anti-inflammatory miRNAs as a result form the first LPS exposure).

Our lab demonstrated that endotoxin tolerance exists in placental tissues (109) and we proposed a model of miRNA-mediated placental immune tolerance packaged within EVs. An LPS stimulus primes placental trophoblasts to exhibit a proinflammatory response through the TLR4 pathway, which activates NF-κB to increase pro-inflammatory cytokine expression and release (such as TNFα) (Figure 1). Additionally, TLR4 activation increases the production of placenta-specific miRNAs (either free or packaged in EVs; Figure 1) likely also mediated by NF-κB (110, 111). Free miRNAs induce a feedback loop to down-regulate TLR4 signaling and alter related downstream processes, including the inflammatory response, as well as inhibiting mRNA translation of TLR receptors and directly attenuate TLR-receptor levels (Figure 2). The secreted EVs translocate to specific target cells/sites, such as placental trophoblasts (autocrine mechanisms), local gestational tissues (paracrine mechanisms), or other maternal and fetal compartments, via circulating peripheral blood or other extracellular fluid, according to specific chemo-attractants or cell-surface markers. EVs then interact with target tissue using cognate surface markers and release their contents, thereby potentially affecting several biological mechanisms, including protein biosynthesis and/or post-transcriptional regulation. The miRNAs within the EVs will reduce the ability of the target cells to produce TNFα in response to LPS exposure. The anti-inflammatory miRNAs implicated in endotoxin tolerance likely act using similar mechanisms, including negative feedback loop at the TLR receptor, to inhibit downstream regulators of the TLR pathway as well as decrease the transcription of pro-inflammatory molecules, such as TNFα (Figure 2).

**Figure 3:**
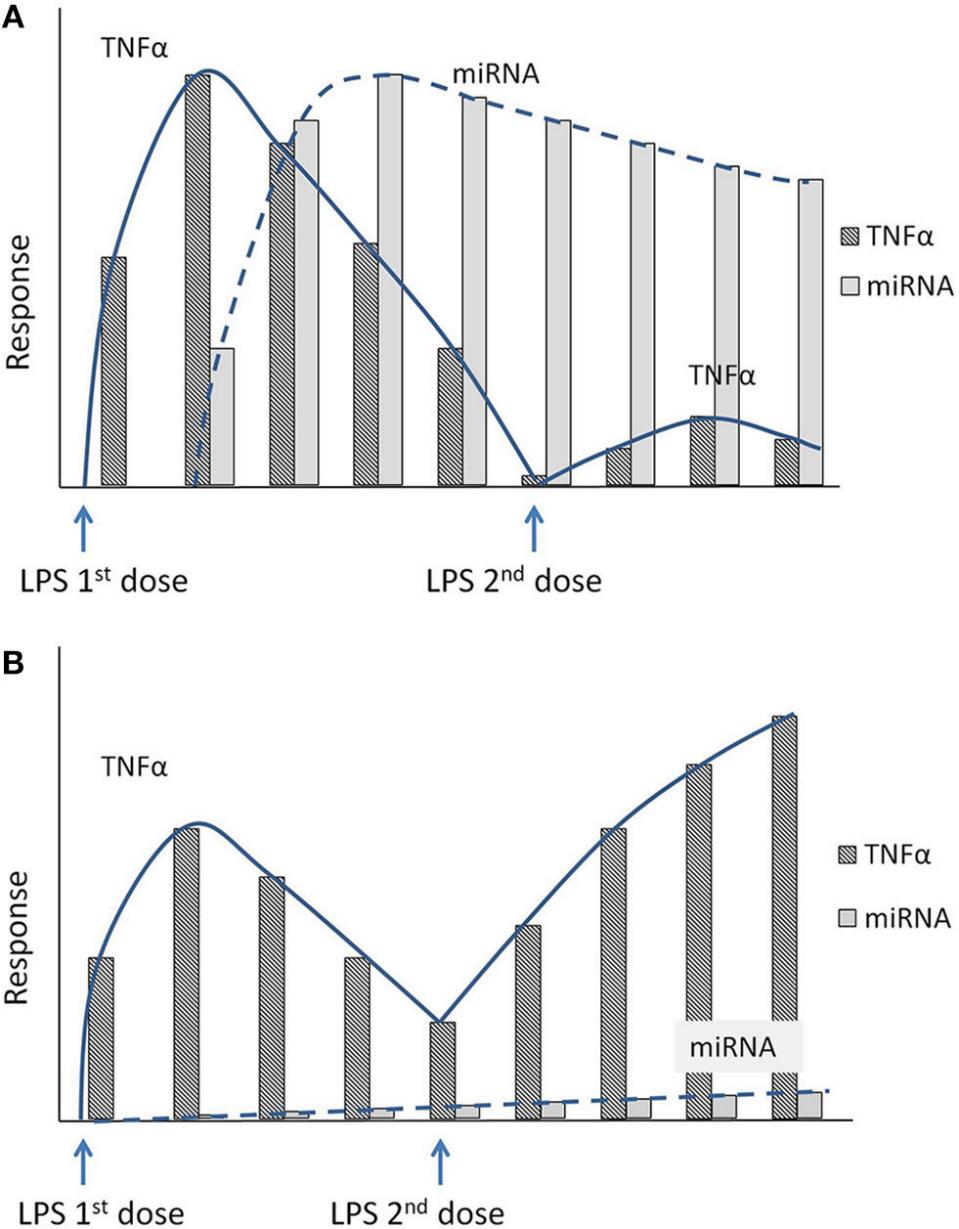
**(A)** Hypothetical figure illustrates the inflammatory response to repeated LPS exposure. Exposure to the first dose of LPS increases the proinflammatory marker TNFα. Shortly following the initial dose of LPS, the cells also secrete specific anti-inflammatory miRNAs that accumulate in the cytoplasm. Upon exposure to second dose of LPS, the sustained high levels of the anti-inflammatory miRNAs will blunt the inflammatory response with decreased TNFα levels (endotoxin tolerance). **(B)** Hypothetical figure illustrates the inflammatory response in the absence of the anti-inflammatory miRNAs mediated endotoxin tolerance. In a knockout model of specific miRNA, the miRNA levels do not increase after initial exposure to LPS. A subsequent exposure to a second dose of LPS thus produces an unchecked inflammatory response with an exaggerated increase in proinflammatory cytokines (TNFα).

Therefore, LPS insult of target cells/tissue harboring these anti-inflammatory miRNAs increases the readiness for subsequent LPS doses by attenuating the TLR response, thereby tipping the balance against a proinflammatory environment at the maternal-fetal interface (immune tolerance to repeated LPS dose). Figure 3A, shows a hypothetical response involving suppressed TNFα levels in the presence of miRNA-induced endotoxin tolerance. Unprimed cells challenged with LPS produced a prominent TNFα response followed by an increase in anti-inflammatory miRNA production, which corresponds to later down-regulation of TNFα (Figure 3A). As shown in Figure 3A, in the presence of sustained high anti-inflammatory miRNA expression, a second dose of LPS will lead to a muted TNFα production by the cells (*tolerized cells*). However, in the absence of these specific miRNAs, subsequent exposure to LPS would result in failure of immune tolerance to repeated infection resulting in exaggerated inflammation at the maternal-fetal interface (Figure 3B). We speculate that placentas from women with infection-induced preterm births will have reduced expression of specific placental anti-inflammatory miRNAs. This will lead to endotoxin tolerance failure and exaggerated inflammatory response to repeated infections that result in preterm births or other inflammatory diseases of pregnancy.

## Concluding Remarks

In summary, we propose that miRNAs play a vital role in mediating immune tolerance at the maternal-fetal interface by attenuating immune responses following repeated exposure to inflammatory insult. Packaging and transport of miRNAs by EVs is suggested as a “smart” process specifically intended to address the potential requirements of the target cell/tissue. The success of this activity is dependent upon the homeostasis of the origin cell (placenta, in this case). Therefore, normal or pathologic conditions in the placenta might affect EVs composition and number, resulting in an altered response to endotoxins or other inflammatory stimuli. Additionally, we suggest that the target cells are not randomly selected and are, in fact, pre-identified according to specific markers presented on the EV surface to allow specific identification of target cell/tissue. Perturbations in this process can result in the failure of miRNA-mediated endotoxin tolerance and an imbalanced proinflammatory state leading to adverse pregnancy outcomes, including preterm labor or preeclampsia. The identification of placenta-specific miRNAs and EV markers will promote identification of novel molecules as potential biomarkers for further study of endotoxin tolerance, as well as possible molecular targets for controlling injury from failed immune tolerance.

## Author Contributions

All authors listed have made a substantial, direct and intellectual contribution to the work, and approved it for publication.

### Conflict of Interest Statement

The authors declare that the research was conducted in the absence of any commercial or financial relationships that could be construed as a potential conflict of interest.

## Abbreviations

CD: cluster of differentiation
DAMP: damage associated molecular patterns
EV: extracellular vesicle
IL: interleukin
IRAK: interleukin-1 receptor associated kinase
miRNA: microRNA
MV: microvesicles
MVB: multivesicular body/late endosome
MyD88: myeloid differentiation primary response 88 protein
NF-κB: transcription factor nuclear factor kappa-light-chain-enhancer of activated B cells
PAMP: pathogen associated molecular patterns
PLAP: placental alkaline phosphatase
PRR: pattern recognition receptors
TLR: toll-like receptor
TNF: tumor necrosis factor
TRAF6: TNF-receptor associated factor 6.
